# Changes in optic nerve head microvasculature following disc hemorrhage absorption in glaucomatous eyes

**DOI:** 10.1038/s41598-025-86460-7

**Published:** 2025-02-01

**Authors:** Jooyoung Yoon, Kyung Rim Sung, Ko Eun Kim, Hyo Ji Han, Joon Mo Kim

**Affiliations:** 1 Department of Ophthalmology, Hanyang University Guri Hospital, Hanyang University College of Medicine, Guri, Republic of Korea; 2 Department of Ophthalmology, Asan Medical Center, University of Ulsan College of Medicine, 88 Olympic-ro 43-gil, Songpa-gu, Seoul, 05505 Republic of Korea; 3 Department of Ophthalmology, Kangbuk Samsung Hospital, Sungkyunkwan University School of Medicine, Seoul, Republic of Korea

**Keywords:** Diseases, Risk factors

## Abstract

This study investigated the changes in optic nerve head (ONH) microvasculature, circumpapillary retinal nerve fiber layer (cpRNFL) thickness, and visual field (VF) sensitivity following the absorption of optic disc hemorrhage (DH). Intradisc vessel density (dVD) was calculated using a 3 × 3 mm optic disc scan in 60 eyes of 60 patients with primary open angle glaucoma and DH who had undergone two or more swept-source optical coherence tomography angiography exams. Clinical parameters at the time of DH occurrence and after absorption, as well as those between the subgroups based on DH recurrence and location, were compared. Linear regression analysis was performed to identify factors associated with changes in cpRNFL thickness in the DH-affected quadrant. Mean dVD, cpRNFL thickness, and VF sensitivity significantly decreased after DH absorption (all *P* < 0.05). The reduction in dVD was more pronounced in eyes with recurrent DH compared to those with a single episode (*P* = 0.032). Eyes with DH occurring within or at the margin of the disc cup showed a greater dVD reduction than those with DH occurring outside the disc cup (*P* = 0.049). The reduction in cpRNFL thickness in the DH-affected quadrant correlated with dVD reduction in the same quadrant (*β* = 0.370, *P* = 0.013) and DH recurrence (*β* = -2.617, *P* = 0.033). This finding suggests that DH pathogenesis may be associated with changes in optic disc vasculature, contributing to glaucomatous progression.

## Introduction

Optic disc hemorrhage (DH) is well-recognized as a significant risk factor for both the development^1^ and progression^2–5^ of glaucoma, yet its underlying pathogenesis remains undetermined. Some studies propose that vascular insufficiency plays a pivotal role in both glaucoma and DH^6^ and suggest that DH is indicative of vasculopathogenic events within the optic nerve head (ONH)^6^, signifying ischemic damage to the ONH^6,7^. Conversely, other studies regard DH as a consequence of structural disruption in the ONH. Specifically, vascular damage may result from the mechanical collapse of the ONH structure^8^, posterior migration of the lamina cribrosa (LC)^9^, or microvascular disruption due to the rapid degeneration of the retinal nerve fiber layer (RNFL) or neuroretinal rim^10^. Thus, DH can arise from either vascular or structural glaucomatous damage to the ONH.

Given the significant association between DH and glaucomatous damage, DH serves as a potential indicator for further structural and functional progression in glaucoma. Previous studies have demonstrated the spatial correlation of DH with alterations in ONH anatomy^11^, peripapillary blood flow^12^, neuroretinal rim notching^13^, progressive localized RNFL thinning^14–16^, and corresponding visual field (VF) deterioration^5,10^. Our group has observed lower intradisc vessel density (dVD) in glaucomatous eyes with DH compared to those without^11^. Additionally, eyes with DH and reduced dVD have shown faster glaucomatous progression^17^, indicating that DH and lower dVD may serve as markers for increased risk of progression. Therefore, assessing dynamic changes in ONH microvasculature after DH occurrence is crucial for understanding the pathophysiologic process underlying structural and functional progression, as well as for predicting progression.

In light of these findings, we investigated alterations in dVD, circumpapillary retinal nerve fiber layer (cpRNFL) thickness, and VF sensitivity following the absorption of DH. We analyzed factors influencing subsequent changes in cpRNFL thickness specifically within the DH-affected sector. Furthermore, we classified patients into subgroups based on the recurrence and location of DH, comparing these subgroups in terms of changes in clinical parameters, including ONH microvasculature.

## Methods

### Study participants

This study was approved by the Institutional Review Board (IRB) of Asan Medical Center (No. 2022-1517) and adhered to the tenets of the Declaration of Helsinki. Informed consent from the study subjects was waived by the IRB of Asan Medical Center due to the retrospective study design.

Patients who visited the glaucoma clinic of Asan Medical Center between May 2020 and June 2023 were retrospectively reviewed. Study participants underwent comprehensive eye examinations, including best-corrected visual acuity, intraocular pressure (IOP) measurement with Goldmann applanation tonometry, slit-lamp biomicroscopy, and gonioscopy. Participants also received swept-source optical coherence tomography angiography (SS-OCTA) (version 2.0.1.47652; PLEX Elite 9000; Carl Zeiss Meditec, Inc., Dublin, CA, USA), spectral-domain optical coherence tomography (SD-OCT) (version 11.5.2.54532; Cirrus high-definition optical coherence tomography [HD-OCT]; Carl Zeiss Meditec, Inc.), dilated color fundus photography (Canon, Tokyo, Japan), ONH stereoscopic photography, red-free RNFL photography (Canon), and Humphrey Field Analyzer Swedish Interactive Threshold Algorithm (SITA) 24-2 VF testing (Carl Zeiss Meditec, Inc.). During the follow-up period, patients had serial ONH and red-free RNFL photography, SS-OCTA, SD-OCT, and standard 24-2 VF tests at regular 4- to 6-month intervals. The results during DH, and the first results after DH absorption were analyzed.

Participants with glaucomatous damage and DH who met the inclusion criteria were enrolled in the study. Inclusion criteria were: age > 18 years, open-angle by gonioscopy, a spherical equivalent between − 6.0 and + 3.0 diopters (D), and cylinder correction within + 3.0 D. Participants had more than 3 years of follow-up with regular optic disc, optical coherence tomography, and VF exams at 4- to 6-month intervals. Exclusion criteria included congenital glaucoma, glaucoma due to secondary causes, other intraocular diseases (e.g., age-related macular degeneration, diabetic retinopathy, non-glaucomatous optic neuropathy), and a history of ocular interventions (except for uncomplicated cataract surgery).

Glaucoma was diagnosed based on the presence of glaucomatous optic disc changes such as optic disc rim thinning, notching, disc excavation, or RNFL defects with compatible VF damage. Glaucomatous VF defects were defined by at least two of the following criteria: (1) a glaucoma hemifield test beyond normal limits, (2) a pattern standard deviation with a probability of < 5%, and (3) a pattern deviation plot containing at least three abnormal points depressed to *P* < 5%, including at least one of these points depressed to *P* < 1%^18^.

DH was identified as an isolated splinter-like or flame-shaped hemorrhage on the optic disc or peripapillary area^19,20^. Recurrent DH was determined by reviewing previous follow-up exams and was defined as the occurrence of an additional DH in the same or a different location within the past three years^11,19,20^. The location of DH was described in terms of the quadrant and proximity to the disc center. When the DH was located over two adjacent quadrants, it was considered to be located in the quadrant with greatest involvement. Eyes with DH were categorized into two groups based on the proximal location of DH:^21^ the LC or cup margin group (proximal DH group) versus the disc rim or peripapillary group (distal DH group). DH was considered unrelated to glaucoma if associated with conditions such as papillitis, ischemic optic neuropathy, diabetic retinopathy, retinal vein occlusion, and acute posterior vitreous detachment at the time of DH, and these cases were excluded from the analysis. The presence, absorption, and location of the DH were independently evaluated by two glaucoma specialists (J.Y. and K.R.S.), who were masked to the patients’ clinical information and test results. Discrepancies between observers were resolved by consensus.

### dVD assessment

**Fig. 1:**
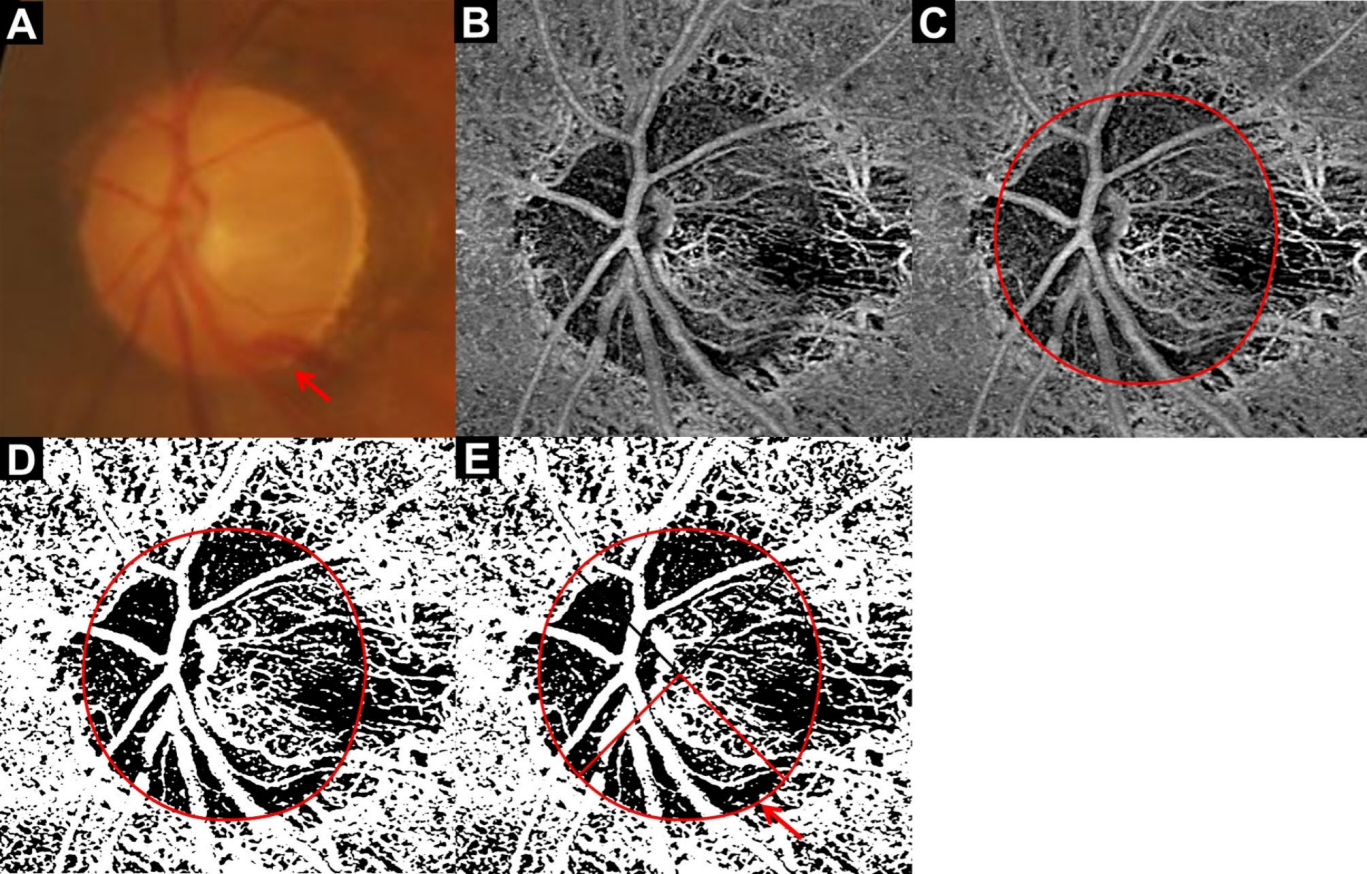
Measurement of the intradisc vessel density (dVD). (**A**) Color disc image. (**B**) En-face whole signal mode optic nerve head (ONH) optical coherence tomography angiography (OCTA) image. (**C**) Same image as (**B**) with the disc margin demarcated by the red line. (**D**) En-face OCTA image of ONH, converted to binary format. The dVD was calculated as the percentage of the white vascular area within the optic disc. (**E**) Same image as (**D**) with the ONH area divided into four sectors (superior, temporal, inferior, and nasal) according to a 90° range around the ONH center. Since disc hemorrhage (DH) was observed in the inferior qudarant (red arrow, **A**), the dVD of the inferior quadrant was considered as the dVD in the DH-affected quadrant (wedge shape demarcated with red line and accompanied by a red arrow, **E**).

The optic disc was scanned using the whole signal mode 3.0 × 3.0-mm ONH image obtained via SS-OCTA. Images with a signal strength of 7 or higher were included, and the built-in FastTrac motion correction software (Carl Zeiss Meditec, Inc.) was applied to minimize motion artifacts. The dVD was measured from the 3.0 × 3.0-mm en face ONH images, as previously described^11,17^. After importing the en face ONH images into the ImageJ software (version 1.54f; National Institutes of Health, Bethesda, MD, USA; http://imagej.nih.gov/ij/), the optic disc boundary was manually outlined (Fig. 1A–C). The images were converted into black-and-white binary images using the mean threshold algorithm, with the threshold set at the mean of the gray level (Fig. 1D). The vascular region appeared as a white area in the image, and the dVD was calculated as the percentage of the white region within the total ONH area. dVD was measured for the entire ONH and in four sectors (superior, temporal, inferior, and nasal) based on a 90° range around the ONH center (Fig. 1E). All measurements were performed by two independent investigators who were masked to the patients’ clinical information and test results (J.Y. and K.R.S) and averaged data were analyzed.

### cpRNFL thickness assessment

The cpRNFL thickness was measured using SD-OCT. Only images with a signal strength of ≥ 7 and without segmentation errors were included. The cpRNFL thicknesses for the overall area and four sectors (superior, temporal, inferior, and nasal), automatically calculated by the internal software, were evaluated in the analyses.

### VF sensitivity assessment

All VF tests were performed with the Humphrey Field Analyzer using the 24-2 SITA protocol. Reliable tests were defined as having a fixation loss of < 20%, a false positive rate of < 33%, and a false negative rate of < 33%^22^. Of the 54 test points of the 24-2 test pattern, 2 points of physiologic scotoma were excluded, and the total threshold values of the remaining 52 points were recorded. Sectoral evaluation was conducted according to the Garway–Heath distribution map^23^, assigning VF test points to corresponding ONH quadrants.

### Statistical analysis

The normality of distribution was assessed using the Kolmogorov–Smirnov test. The intraclass correlation coefficient was calculated to assess the extent of inter-observer agreement between two graders (J.Y. and K.R.S) for dVD measurement. Parameters at the time of and after DH absorption were compared using paired *t*-tests. Factors associated with the change in cpRNFL thickness in the DH-affected quadrant were evaluated using linear regression analysis. In addition to dVD and DH characteristics, the covariates of the analysis were selected based on the factors that could influence the structural and vascular status of the LC. Parameters affecting the LC structure, such as age^24^, IOP^25^, and axial length^26^, as well as underlying systemic vascular diseases—including hypertension, diabetes mellitus, and hyperlipidemia—that could induce hemodynamic instability^27,28^ were included. Factors known to be associated with glaucoma progression, such as age^29–31^, male gender^31^, IOP^29,30^, central corneal thickness^30^, and VF measures as the standard indicators of glaucoma severity^29,31^, were also evaluated as potential confounding factors. Factors with *P* < 0.05 in the univariable analysis were included in the multivariable analysis. Clinical parameters were compared between the two subgroups based on DH recurrence and location using independent *t*-tests. Pearson correlation analysis was applied to assess the correlation between two parameters. All statistical analyses were performed using SPSS (version 22.0; SPSS Inc., Chicago, IL, USA). A *P*-value of < 0.05 was considered statistically significant.

## Results

A total of 67 eyes from 67 patients with primary open angle glaucoma who experienced at least one episode of DH and met the inclusion criteria were enrolled in the study. Seven eyes were excluded due to poor-quality SS-OCTA images. Of the remaining 60 eyes, 8 eyes (13.3%) exhibited DH at the LC, 22 (36.7%) at the optic disc cup margin (proximal group), 24 (40.0%) at the disc rim, and 6 (10.0%) in the peripapillary area (distal group) (Table 1). DH was most prevalent in the inferior quadrant (33 eyes, 55.0%), followed by the superior quadrant (18 eyes, 30.0%) and the temporal quadrant (9 eyes, 15.0%). No eyes exhibited DH in multiple quadrants, and recurrent DH was observed in 32 eyes (53.3%). The mean interval between imaging at the time of DH and its absorption was 9.9 ± 2.4 months (range 6.9–12.8 months). The inter-observer agreement for dVD measurement was excellent, with an intraclass correlation coefficient of 0.912 (95% confidence interval [CI] [0.908, 0.915]).

**Table 1:** Demographics of primary open-angle glaucoma patients with disc hemorrhage (DH).

Variables	Entire cohort (*n* = 60)
Age, years	59.3 ± 11.2
Male/female, n	39/21
Test interval, months	9.9 ± 2.4
Intraocular pressure at the time of DH, mmHg	14.1 ± 2.7
Central corneal thickness, µm	538.76 ± 39.22
Axial length, mm	24.79 ± 1.51
Family history of glaucoma, n (%)	6 (10.0%)
Hypertension, n (%)	16 (26.7%)
Diabetes mellitus, n (%)	12 (20.0%)
Dyslipidemia, n (%)	9 (15.0%)
DH site	
Superior, n (%)	18 (30.0%)
Temporal, n (%)	9 (15.0%)
Inferior, n (%)	33 (55.0%)
Nasal, n (%)	0 (0.0%)
DH location	
Proximal, n (%)	30 (50.0%)
Lamina cribrosa, n (%)	8 (13.3%)
Cup margin, n (%)	22 (36.7%)
Distal, n (%)	30 (50.0%)
Disc rim, n (%)	24 (40.0%)
Peripapillary, n (%)	6 (10.0%)

The mean dVD and cpRNFL thickness significantly decreased after DH absorption (all, *P* < 0.05) (Table 2). Most sectoral values showed similar trends, with the greatest reduction observed in the DH-affected quadrant. Although the VF mean deviation (MD) and visual field index (VFI) did not show significant changes, the mean, superior, and affected sector VF sensitivity significantly decreased after DH absorption.

**Table 2:** Comparison of changes in intradisc vessel density, circumpapillary retinal nerve fiber layer thickness, and visual field between at the time of disc hemorrhage (DH) and post-DH.

Variables	Entire cohort (*n* = 60)		
	At the time of DH	After DH	*P*
Intradisc vessel density, %			
Mean	50.12 ± 3.48	48.83 ± 3.64	**0.003**
Superior	48.35 ± 6.00	47.56 ± 5.80	0.147
Temporal	44.01 ± 8.20	42.03 ± 8.55	**0.005**
Inferior	53.08 ± 5.56	51.14 ± 6.60	**0.001**
Nasal	48.26 ± 6.63	47.66 ± 6.88	0.351
DH-affected sector	50.09 ± 6.32	48.07 ± 7.45	**< 0.001**
Circumpapillary retinal nerve fiber layer thickness, µm			
Mean	76.51 ± 10.28	74.09 ± 9.92	**< 0.001**
Superior	93.87 ± 17.91	90.27 ± 16.34	**< 0.001**
Temporal	65.49 ± 12.19	62.44 ± 10.59	**< 0.001**
Inferior	83.60 ± 18.60	81.40 ± 19.09	**0.004**
Nasal	62.71 ± 8.53	62.24 ± 10.17	0.568
DH-affected sector	82.67 ± 17.95	79.29 ± 17.83	**< 0.001**
Visual field			
Mean deviation, dB	-3.70 ± 3.74	-4.03 ± 4.01	0.149
Visual field index, %	91.17 ± 11.12	90.42 ± 12.46	0.227
Visual field sensitivity, dB			
Mean	26.77 ± 3.59	26.36 ± 3.77	**0.022**
Superior	25.45 ± 5.71	24.94 ± 6.08	**0.018**
Temporal	29.20 ± 3.80	28.82 ± 4.50	0.248
Inferior	27.14 ± 4.06	26.74 ± 4.53	0.086
Nasal	28.06 ± 2.47	28.17 ± 3.23	0.746
DH-affected sector	26.49 ± 3.82	25.96 ± 4.71	**0.030**

Values are shown in mean ± standard deviation.

Statistically significant values are indicated in bold.

In the univariable analysis, the reduction in cpRNFL thickness in the DH-affected sector was linearly related to the mean (*β* = 0.451, 95% CI [0.043, 0.858], *P* = 0.031) and affected (*β* = 0.439, 95% CI [0.138, 0.741], *P* = 0.005) sector dVD reduction, recurrence of DH (*β* = -3.350, 95% CI [-5.662, -1.038], *P* = 0.005), and the proximal location of DH to the ONH center (*β* = 2.673, 95% CI [0.667, 5.217], *P* = 0.012) (Table 3). Two separate multivariable analyses were conducted to avoid multicollinearity between mean and affected sector dVD reduction (*r* = 0.642, *P* < 0.001). The affected sector dVD reduction (*β* = 0.370, 95% CI [0.083, 0.657], *P* = 0.013) and recurrence of DH (*β* = -2.617, 95% CI [-5.016, -2.017], *P* = 0.033) were independently associated with cpRNFL thickness reduction after adjusting for other variables.

**Table 3:** Comparisons of clinical parameters between recurrent and single-episode disc hemorrhage (DH) groups.

		Univariable analysis			Multivariable analysis – 1			Multivariable analysis – 2		
		*β*	95% CI	*P*	*β*	95% CI	*P*	*β*	95% CI	*P*
Age		-0.001	-0.110, 0.108	0.980						
Male		0.485	-2.160, 3.130	0.715						
Intraocular pressure at the time of DH		0.039	-0.433, 0.512	0.868						
Central corneal thickness		0.007	-0.027, 0.042	0.669						
Axial length		-0.377	-1.326, 0.571	0.427						
Hypertension		-0.431	-3.165, 2.302	0.753						
Diabetes mellitus		0.477	-2.627, 3.581	0.759						
Dyslipidemia		1.324	-2.181, 4.830	0.452						
At the time of DH	Mean dVD	-0.016	-0.369, 0.337	0.928						
	DH-affected sector dVD	0.052	-0.143, 0.247	0.595						
	Mean VF sensitivity	0.058	-0.294, 0.409	0.742						
	DH-affected sector VF sensitivity	0.090	-0.236, 0.417	0.582						
Difference between at the time of DH and post-DH	Mean dVD	0.451	0.043, 0.858	**0.031**	0.389	0.010, 0.789	0.069			
	DH-affected sector dVD	0.439	0.138, 0.741	**0.005**				0.370	0.083, 0.657	**0.013**
	Mean VF sensitivity	0.774	-0.197, 1.745	0.116						
	DH-affected sector VF sensitivity	0.634	-0.066, 1.333	0.075						
Recurrence of DH		-3.350	-5.662, -1.038	**0.005**	-2.617	-5.016, -0.217	**0.033**	-1.973	-4.453, 0.507	0.116
Proximal location of DH		2.673	0.667, 5.217	**0.012**	0.739	-0.589, 4.067	0.140	1.949	-0.288, 4.186	0.086

Statistically significant values are indicated in bold.

CI = confidence interval; dVD = intradisc vessel density; VF = visual field.

The recurrent DH group showed significant dVD reduction after DH absorption in the mean, temporal, inferior, and affected sectors, whereas the single-episode DH group did not show such changes. The reduction of dVD after DH absorption was significantly greater in the recurrent DH group in the inferior and affected sectors (*P* = 0.049 and *P* = 0.032, respectively) (Table 4). The change in cpRNFL thickness also differed significantly in the inferior and DH-affected sectors in the recurrent DH group compared with the single episode DH group (*P* = 0.004 and *P* = 0.005, respectively). VF sensitivity decreased in the mean, superior, temporal, and affected sectors only in the recurrent DH group (*P* = 0.020, *P* = 0.011, *P* = 0.044, and *P* = 0.029, respectively). The mean interval between examinations was 10.1 ± 2.6 months in the recurrent DH group and 9.6 ± 2.2 months in the single DH group, showing no significant difference (*P* = 0.641).

**Table 4:** Comparisons of clinical parameters between recurrent and single-episode disc hemorrhage (DH) groups.

	Recurrent DH group (*n* = 32)			Single-episode DH group (*n* = 28)			P**		
	At the time of DH	After DH	*P* ^*^	At the time of DH	After DH	P*	At the time of DH	After DH	Difference
Intradisc vessel density, %									
Mean	49.31 ± 2.74	47.71 ± 3.85	**0.006**	51.04 ± 4.03	50.12 ± 2.95	0.156	0.054	**0.009**	0.411
Superior	47.01 ± 5.30	46.22 ± 5.72	0.296	49.89 ± 6.47	49.09 ± 5.61	0.328	0.064	0.056	0.949
Temporal	43.24 ± 8.18	40.68 ± 8.34	**0.011**	44.89 ± 8.29	43.58 ± 8.67	0.188	0.440	0.193	0.368
Inferior	51.97 ± 5.11	48.97 ± 6.98	**0.001**	54.35 ± 5.87	53.62 ± 5.23	0.315	0.098	**0.006**	**0.049**
Nasal	47.83 ± 6.22	47.19 ± 7.33	0.558	48.75 ± 7.15	48.19 ± 6.42	0.373	0.600	0.581	0.949
DH-affected sector	49.70 ± 5.36	46.61 ± 7.57	**< 0.001**	50.53 ± 7.34	49.74 ± 7.08	0.298	0.616	0.106	**0.032**
Circumpapillary retinal nerve fiber layer thickness, µm									
Mean	78.17 ± 8.72	75.28 ± 8.48	**< 0.001**	74.65 ± 11.67	72.77 ± 11.34	**0.001**	0.181	0.533	0.265
Superior	98.31 ± 12.43	92.97 ± 9.99	**0.001**	88.92 ± 21.71	87.27 ± 21.14	0.100	**0.044**	0.286	0.051
Temporal	66.52 ± 12.93	64.03 ± 11.27	**0.004**	64.35 ± 11.46	60.65 ± 9.68	**0.003**	0.450	0.299	0.369
Inferior	83.38 ± 17.53	79.24 ± 17.74	**< 0.001**	83.85 ± 20.07	83.81 ± 20.57	0.972	0.950	0.251	**0.004**
Nasal	65.24 ± 10.41	64.59 ± 8.61	0.606	60.62 ± 8.08	58.89 ± 8.95	0.096	0.081	**0.019**	0.149
DH-affected sector	85.10 ± 14.48	80.14 ± 14.13	**< 0.001**	79.96 ± 21.13	78.35 ± 21.48	0.062	0.293	0.714	**0.005**
Visual field									
Mean deviation, dB	-3.09 ± 3.27	-3.66 ± 2.73	0.103	-4.42 ± 4.17	-4.48 ± 5.16	0.842	0.177	0.441	0.276
Visual field index, %	92.63 ± 8.52	91.72 ± 9.00	0.238	89.44 ± 13.55	88.89 ± 15.66	0.586	0.278	0.390	0.448
Visual field sensitivity, dB									
Mean	27.01 ± 3.36	26.43 ± 3.02	**0.020**	26.47 ± 3.91	26.28 ± 4.59	0.467	0.578	0.888	0.250
Superior	25.04 ± 5.48	24.29 ± 5.55	**0.011**	25.96 ± 6.07	25.75 ± 6.70	0.512	0.555	0.378	0.207
Temporal	29.38 ± 3.69	28.29 ± 4.37	**0.044**	28.98 ± 4.00	29.47 ± 4.66	0.117	0.698	0.335	**0.017**
Inferior	28.10 ± 2.53	27.68 ± 1.92	0.128	25.95 ± 5.21	25.58 ± 6.31	0.353	**0.048**	0.084	0.926
Nasal	28.06 ± 2.88	28.27 ± 2.65	0.629	28.05 ± 1.91	28.04 ± 3.89	0.985	0.983	0.797	0.752
DH-affected sector	26.26 ± 3.28	25.54 ± 3.62	**0.029**	26.78 ± 4.45	26.48 ± 5.82	0.423	0.621	0.462	0.364

*Comparison between at the time and after DH using paired *t*-test.

**Comparison between recurrent and single-episode DH groups using independent *t*-test.

Statistically significant values are indicated in bold.

The proximal DH group (LC + cup margin) showed significant dVD reduction in more sectors and to a greater extent than the distal DH group (disc rim + peripapillary area), including the affected sector (*P* = 0.049) (Table 5). The reduction in cpRNFL thickness was more pronounced in the proximal DH group compared with the distal DH group in the affected sector (*P* = 0.012). Among the proximal DH group, 19 eyes (63.3%) had a history of recurrent DH, while 13 eyes (43.3%) in the distal DH group had recurrent DH, with no statistically significant difference between the two groups (*P* = 0.125).

**Table 5:** Comparisons of clinical parameters between proximal (lamina cribrosa or cup margin) and distal (disc rim or peripapillary) disc hemorrhage (DH) groups.

	Proximal DH group (*n* = 30)			Distal DH group (*n* = 30)			P**		
	At the time of DH	After DH	*P* ^*^	At the time of DH	After DH	P*	At the time of DH	After DH	Difference
Intradisc vessel density, %									
Mean	50.38 ± 3.27	48.26 ± 4.16	**0.003**	49.86 ± 3.72	49.28 ± 2.91	0.248	0.563	0.274	**0.048**
Superior	49.11 ± 5.97	47.81 ± 6.32	0.087	47.60 ± 6.03	47.32 ± 5.36	0.736	0.332	0.752	0.376
Temporal	42.84 ± 8.41	40.46 ± 8.15	**0.030**	45.19 ± 7.96	43.37 ± 8.59	**0.040**	0.271	0.183	0.675
Inferior	52.97 ± 4.63	50.58 ± 6.47	**0.009**	53.20 ± 6.44	51.68 ± 6.80	**0.039**	0.872	0.522	0.505
Nasal	49.31 ± 4.89	47.61 ± 6.56	0.133	47.21 ± 7.95	47.70 ± 7.30	0.421	0.221	0.959	0.086
DH-affected sector	49.52 ± 6.72	46.62 ± 7.90	**< 0.001**	50.66 ± 5.95	49.26 ± 6.74	**0.041**	0.488	0.169	**0.049**
Circumpapillary retinal nerve fiber layer thickness, µm									
Mean	78.86 ± 10.60	76.37 ± 10.46	**< 0.001**	74.59 ± 9.58	72.69 ± 9.77	**0.003**	0.120	0.179	0.223
Superior	97.67 ± 17.01	93.04 ± 14.11	**0.003**	90.28 ± 18.35	87.93 ± 18.60	**0.031**	0.125	0.255	0.131
Temporal	67.26 ± 12.93	64.07 ± 11.16	**0.001**	65.21 ± 12.78	62.00 ± 11.41	**0.004**	0.553	0.495	0.968
Inferior	87.19 ± 19.00	84.19 ± 2.73	**0.005**	79.66 ± 18.22	78.21 ± 17.96	0.171	0.130	0.163	0.308
Nasal	63.08 ± 8.62	63.04 ± 8.89	0.965	62.59 ± 8.72	62.03 ± 11.64	0.678	0.834	0.720	0.716
DH-affected sector	86.12 ± 18.81	81.35 ± 18.96	**< 0.001**	79.10 ± 17.33	77.28 ± 17.33	**0.019**	0.156	0.409	**0.012**
Visual field									
Mean deviation, dB	-4.29 ± 4.63	-4.84 ± 5.03	0.097	-3.13 ± 2.55	-3.26 ± 2.56	0.697	0.236	0.132	0.300
Visual field index, %	89.28 ± 13.95	87.31 ± 16.19	0.054	93.43 ± 7.24	93.00 ± 6.21	0.535	0.201	0.059	**0.049**
Visual field sensitivity, dB									
Mean	25.92 ± 4.61	25.41 ± 4.67	0.081	27.61 ± 1.89	27.32 ± 2.28	0.147	0.079	0.057	0.520
Superior	24.10 ± 7.21	23.42 ± 7.33	**0.028**	26.80 ± 3.28	26.46 ± 4.08	0.271	0.077	0.061	0.418
Temporal	28.09 ± 4.74	27.39 ± 5.49	0.230	30.32 ± 2.10	30.24 ± 2.63	0.824	0.027	**0.017**	0.355
Inferior	26.81 ± 4.61	26.42 ± 5.31	0.327	27.48 ± 3.47	27.06 ± 3.66	0.114	0.542	0.601	0.950
Nasal	27.59 ± 2.80	27.54 ± 3.38	0.923	28.53 ± 2.05	28.79 ± 3.00	0.473	0.158	0.146	0.629
DH-affected sector	26.52 ± 4.32	25.92 ± 4.99	0.089	26.47 ± 3.32	25.92 ± 4.41	0.094	0.965	0.944	0.957

*Comparison between at the time and after DH using paired *t*-test.

**Comparison of proximal and distal DH groups using independent *t*-test.

Statistically significant values are indicated in bold.

**Fig. 2:**
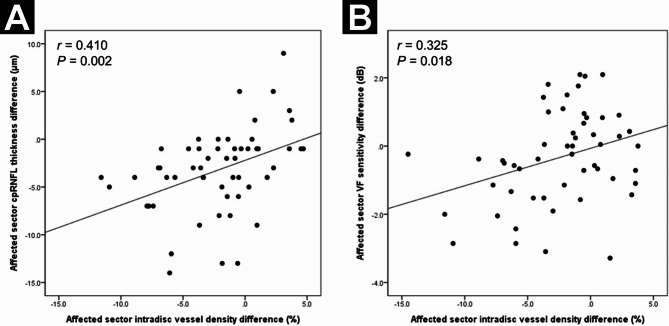
Scatter plots illustrating a significant positive association between the difference in intradisc vessel density in the disc hemorrhage (DH)-affected sector and the difference in circumpapillary retinal nerve fiber layer (cpRNFL) thickness (**A**), as well as visual field (VF) sensitivity (**B**), in the same sector. *r*, Pearson correlation coefficient.

**Fig. 3:**
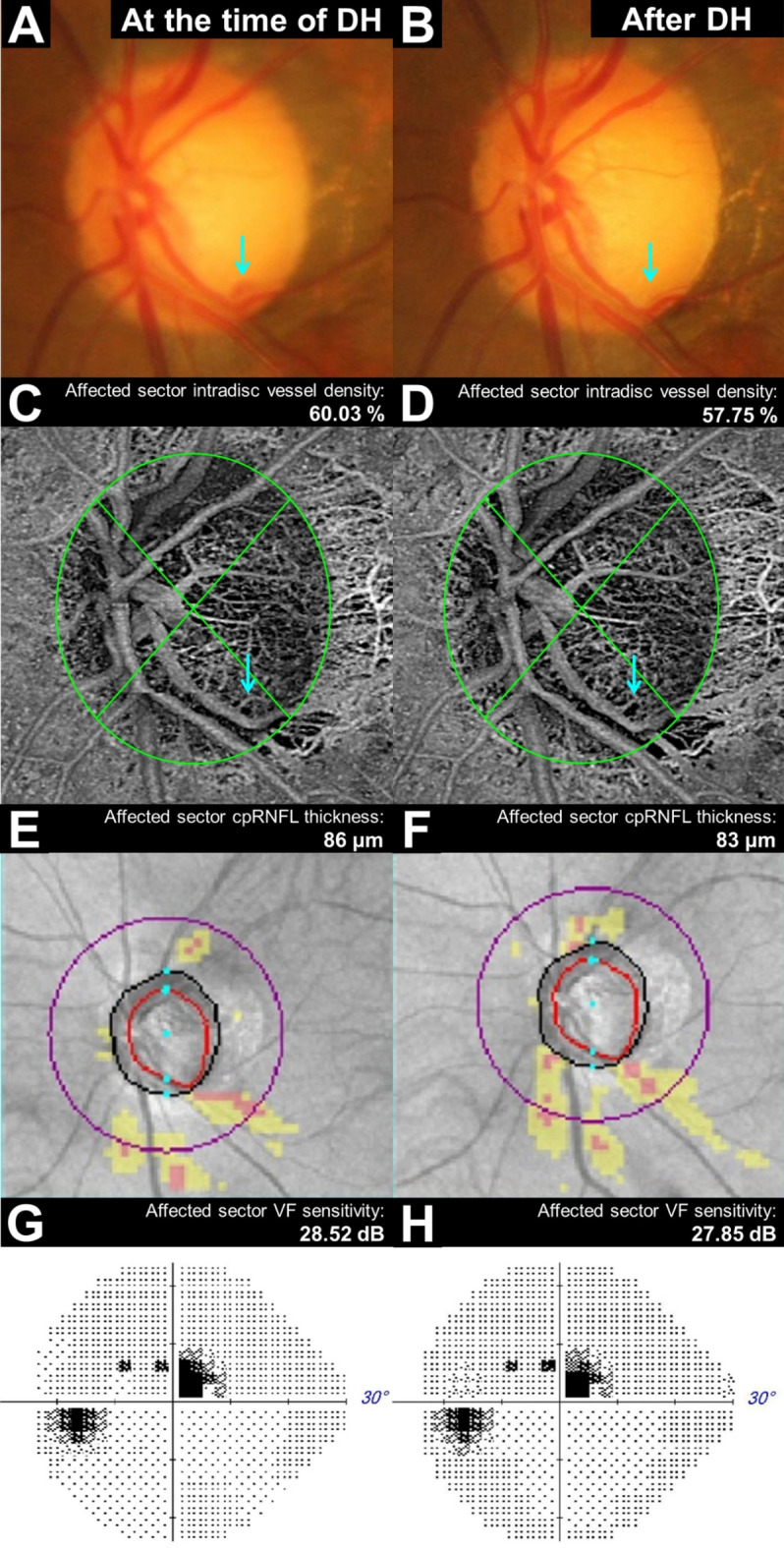
A 64-year-old female with a disc hemorrhage (DH) in the inferotemporal area of her right eye (**A**), which was absorbed after six months (**B**). After DH absorption, the intradisc vessel density in the inferior quadrant reduced from 60.03% (**C**) to 57.75% (**D**), the inferior circumpapillary retinal nerve fiber layer (cpRNFL) thickness decreased from 86 μm (**E**) to 83 μm (**F**), and the superior visual field (VF) sensitivity reduced from 28.52 dB (**G**) to 27.85 dB (**H**).

**Fig. 4:**
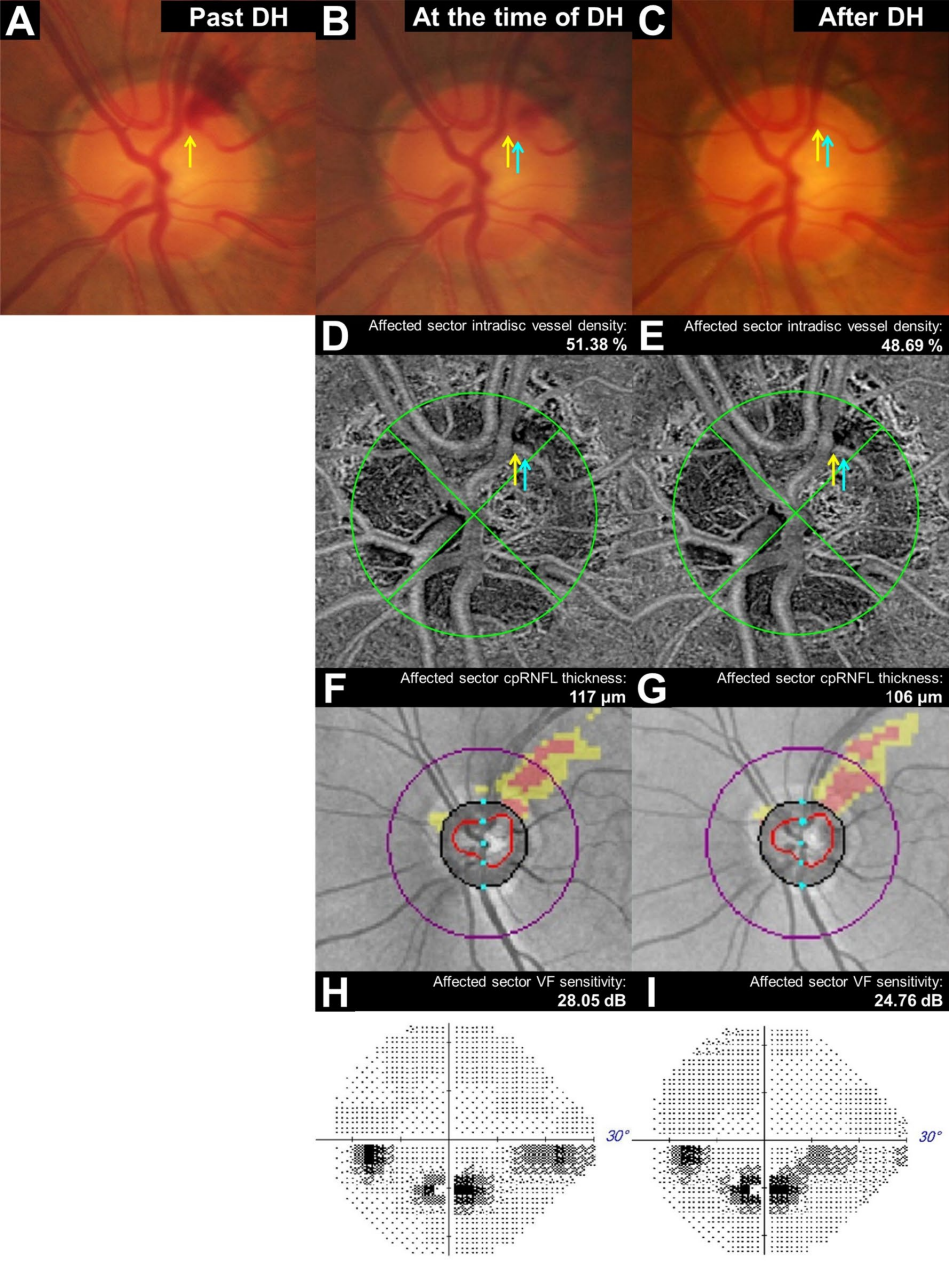
A 57-year-old male with multiple recurrent episodes of disc hemorrhage (DH) in the superotemporal area of his left eye over five years of follow-up. Fundus photos represent previous (**A**), current (**B**), and post-absorption DH (**C**). The intradisc vessel density in the superior quadrant reduced from 51.38% (**D**) to 48.69% (**E**), the superior quadrant circumpapillary retinal nerve fiber layer (cpRNFL) thickness decreased from 117 μm (**F**) to 106 μm (**G**), and the inferior visual field (VF) sensitivity reduced from 28.05 dB (**H**) to 24.76 dB (**I**).

Figure 2 illustrates the linear correlation between dVD and cpRNFL thickness change (*r* = 0.410, *P* = 0.002), and between dVD and VF sensitivity change in the DH-affected sector (*r* = 0.325, *P* = 0.018). Figures 3 and 4 show representative cases of single-episode and recurrent DH, demonstrating significant dVD reduction.

## Discussion

The present study demonstrates that optic disc microvasculature loss occurs after DH absorption and correlates with the subsequent reduction in cpRNFL thickness, as well as with changes in VF sensitivity in the DH-affected sector. The reduction in dVD and cpRNFL thickness was more pronounced in eyes with recurrent DH and those in the proximally located DH group. Since the ONH is the pivotal site of glaucomatous damage^9,32^ and DH represents structural^8–10^/vascular^6,7^ damage within the ONH, this study’s strength lies in its investigation of microvasculature changes within the ONH using high-resolution SS-OCTA in eyes with DH, and its correlation of these changes with other clinical parameters, revealing the association of DH with LC microvasculature and glaucoma progression.

Recent advances in optical coherence tomography angiography (OCTA) technology have enabled the assessment of ONH and peripapillary microvasculature in glaucomatous eyes with DH. Previous studies have shown that a history of DH is linked to decreased peripapillary superficial vessel density (VD)^12,33,34^. Since peripapillary superficial vessels within the RNFL are supplied by retinal circulation, the reduction of peripapillary superficial VD may reflect RNFL thinning. However, the causal relationship between DH and ONH microvasculature has not been fully elucidated. Utilizing high-resolution OCTA to investigate the association between intradisc microvasculature and DH, our study demonstrates that dVD decreases following DH absorption and correlates with RNFL thinning. This contributes to further deterioration in VF sensitivities, particularly in the mean, superior, and DH-affected sectors. Regardless of the cause—whether structural damage induced by posterior migration of the LC^9^, microvascular disruption due to rapid degeneration of the RNFL or neuroretinal rim^10^, or vascular damage caused by mechanical collapse of ONH structure^8^ or primary vascular events such as blood flow stasis or reduced perfusion^35^— these results showed dVD reduction after DH. This phenomenon was more pronounced in cases of recurrent DH, suggesting that recurrent DH is associated with ongoing and more extensive destruction of ONH microvasculature and RNFL. These combined vascular, structural, and functional changes may explain why DH serves as a predictor and indicator for glaucomatous progression.

Although the exact pathogenesis of DH remains unclear, numerous studies have reported decreased perfusion in the ONH area in glaucomatous eyes with DH^11,12,33–35^. Studies evaluating disc fluorescein angiography in eyes with DH and accompanying localized RNFL defects have observed prolongation of arteriovenous transit time and delayed filling or filling defects, suggesting that blood flow stasis and turbulence occur in the ONH during DH^35^. Although this study does not conclusively determine the pathogenesis of DH, it provides valuable insights. By categorizing DH eyes based on the location of DH occurrence, we found that 50% of eyes showed DH at the LC or cup margin (proximal group) and 50% showed DH in the rim or peripapillary area (distal group). The proximal group exhibited greater dVD reduction than the distal group, possibly due to the association between proximally located DH and focal LC defects. The location of DH may indicate the origin of glaucomatous stress in the ONH^21^. Previous studies have indicated that DH with focal LC defects is more proximally located to the disc center^36^, and that glaucomatous VF progression is faster in eyes with DH at the focal LC defect site compared with those without focal LC defects^20^. Thus, proximally located DH is more strongly associated with focal LC defects, implying greater structural damage and future functional impairment. Persistent stress to the LC may cause LC defects and subsequent damage, manifesting as DH. The present study did not analyze the pathophysiologic mechanisms of DH and their association with IOP, which we believe should be investigated in the future.

Recurrent DH is associated with more rapid glaucoma progression. Significantly greater RNFL thinning^16,37^ and notably progressive VF changes^5^ were observed in recurrent DH eyes compared with non-recurrent DH eyes. Some studies have proposed that hemodynamic status and the vascular damage secondary to hemodynamic abnormalities may differ among patients with glaucoma with and without recurrent DH^19,27^. Recent studies revealed that high systolic and diastolic blood pressure^27^ as well as vascular symptoms such as cold extremities, Raynaud’s phenomenon, orthostatic hypotension, or migraine^19^, are more prevalent in individuals with recurrent DH than in those with non-recurrent DH. Moreover, high mean arterial systemic blood pressure reduced macular VD in glaucomatous eyes with DH but not in those without^28^, implying relatively impaired autoregulation of blood flow in eyes with DH, which may cause hemodynamic changes that impact ocular perfusion. Furthermore, a significant decrease in ocular perfusion, presenting as a greater reduction in peripapillary VD^12^ and more prevalent choroidal microvascular dropout^19^ was observed in recurrent DH eyes compared with those without. This study results align with these findings, showing a significantly greater decrease in cpRNFL thickness, VF sensitivity, and dVD in the recurrent DH group compared to the single episode DH group. Although the present study did not evaluate the systemic hemodynamic parameters of the patients, these findings suggest that susceptibility to hemodynamic changes in eyes with recurrent DH may have contributed to more significant optic disc microvasculature loss after DH.

This study has several limitations. First, the frequency of follow-up may have impacted the detection of DH, and some instances of DH that appeared and resolved between follow-ups may have been missed. Such characteristics of DH may commonly impact DH related studies. To minimize undetected DH, only participants with more than 3 years of follow-up and routine optic disc evaluations at 4- to 6-month intervals were included. Second, large vessels within the ONH were included in the VD estimation. Some studies^11,42^ have excluded major retinal vessels when calculating VD inside the ONH to avoid projection artifacts. In contrast to previous studies, this study aimed to evaluate changes within the same participants, assuming that obscuration by the same major vessels would not impact the measurement of changes in optic disc microvasculature. Lastly, projection artifacts caused by DH may have influenced the dVD evaluation. Previous studies have shown that the presence of DH does not affect the measurement of peripapillary VD or cpRNFL thickness^43^, likely due to the even distribution of the relatively small amount of hemorrhage. However, depending on the size or density of DH, dVD at the time of DH may be underestimated if the DH obscures the microvasculature within the ONH. Our study results, showing a significant decrease in dVD after DH, may be enhanced if the potential projection error of DH is removed. Further evaluation is needed to determine whether DH can alter dVD measurements.

In conclusion, ONH microvasculature decreases after DH, with the degree of change and cpRNFL thickness reduction in the DH-affected sector being strongly correlated. The decrease in optic disc microvasculature and cpRNFL thickness was prominent in recurrent DH eyes and in eyes with DH occurring within the optic disc cup. Hence, eyes with recurrent DH or proximally located DH require meticulous observation and management due to their tendency of rapid glaucomatous structural and functional progression.

## Data Availability

Data are available upon reasonable request. The datasets generated and analyzed during the current study are available from the corresponding author on reasonable request.
